# RETRACTION: Prevalence of Pressure Ulcer and Related Factors in Orthopaedic Wards: A Systematic Review and Meta‐Analysis

**DOI:** 10.1111/iwj.70245

**Published:** 2025-02-25

## Abstract

**RETRACTION:** AsadiK. , FouladpourA. , VajargahP. G. , MollaeiA. , FiroozM. , HosseiniS. J. , MahdiabadiM. Z. , SamidoustP. , TakasiP. , KarkhahS. , SalariA. , and ArisA. , “Prevalence of Pressure Ulcer and Related Factors in Orthopaedic Wards: A Systematic Review and Meta‐Analysis,” International Wound Journal 20, no. 7 (2023): 2914–2923, 10.1111/iwj.14156.36960790 PMC10410314

The above article, published online on 24 March 2023, in Wiley Online Library (http://onlinelibrary.wiley.com/), has been retracted by agreement between the journal Editor in Chief, Professor Keith Harding; and John Wiley & Sons Ltd. A third party reported to the journal that they had found evidence of excessive self‐citations in the reference list of this article. The publisher did not confirm the evidence of excessive self‐citations. Upon further investigation, the publisher also concluded that this article was accepted solely on the basis of a compromised peer review process. In view of the clear evidence of compromised peer review, the parties agreed that the paper must be retracted. The authors disagree with the retraction.

**RETRACTION:** K. Asadi , A. Fouladpour , P. G. Vajargah , A. Mollaei , M. Firooz , S. J. Hosseini , M. Z. Mahdiabadi , P. Samidoust , P. Takasi , S. Karkhah , A. Salari , and A. Aris , “Prevalence of Pressure Ulcer and Related Factors in Orthopaedic Wards: A Systematic Review and Meta‐Analysis,” International Wound Journal 20, no. 7 (2023): 2914–2923, 10.1111/iwj.14156.36960790 PMC10410314

The above article, published online on 24 March 2023, in Wiley Online Library (http://onlinelibrary.wiley.com/), has been retracted by agreement between the journal Editor in Chief, Professor Keith Harding; and John Wiley & Sons Ltd. A third party reported to the journal that they had found evidence of excessive self‐citations in the reference list of this article. The publisher did not confirm the evidence of excessive self‐citations. Upon further investigation, the publisher also concluded that this article was accepted solely on the basis of a compromised peer review process. In view of the clear evidence of compromised peer review, the parties agreed that the paper must be retracted. The authors disagree with the retraction.

